# Soluble osteopontin concentrations in serum and ascites of women with advanced serous ovarian cancer

**DOI:** 10.1186/2050-6511-13-S1-A72

**Published:** 2012-09-17

**Authors:** Katarina Černe, Ana Bačnik, Katarina Galič-Jerman, Borut Kobal

**Affiliations:** 1 Institute of Pharmacology and Experimental Toxicology, Faculty of Medicine, University of Ljubljana, 1000 Ljubljana, Slovenia; 2 Division of Gynaecology, University Medical Centre Ljubljana, 1000 Ljubljana, Slovenia

## Background

Despite advances in surgery and combination chemotherapy, ovarian cancer is first in terms of death rates of gynaecological malignancies. More than 90% of ovarian cancers arise from surface epithelium and the serous histological subtype is the most commonly diagnosed epithelial ovarian carcinoma. Extensive seeding of the peritoneal cavity by tumour cells is often associated with ascites, particularly in advanced, high-grade serous carcinomas. Currently CA-125 is the most widely used biomarker in the evaluation and management of women with epithelial ovarian cancer. However, in approximately 15% of these patients CA-125 is not indicative of disease status or progression. Therefore, an alternative tumour marker would be useful. Osteopontin is a secreted, integrin-binding glykophosphoprotein which is overexpressed in ovarian cancer cells and thus may serve as a serum biomarker. By combining the data from blood and fluid from the proximity of the tumour we might be more likely to discover a protein biomarker secreted from the tumour rather than deriving from another part of the body.

## Methods

We analysed twenty patients treated at the Department of Gynaecology, Ljubljana, divided into two groups: controls (without adnexal pathology) and patients with advanced serous ovarian cancer (International Federation of Gynecology and Obstetrics (FIGO) stage III and IV). Both serum and free peritoneal fluid including ascites were collected and examined. Preoperative osteopontin concentrations were determined using the FlowCytomix Simplex kit (eBioscience). FlowCytomix Pro 2.4 (eBioscience) was used for data analysis.

## Results

Patients with advanced ovarian cancer had significantly increased serum osteopontin concentration vs. controls (p < 0.013) and increased concentration of osteopontin in ascites vs. peritoneal fluid from control patients (p < 0.001).

## Conclusions

Our preliminary results suggest that osteopontin might represent an effective biomarker associated with advanced serous ovarian cancer due to its elevated levels in both serum and ascites. The potential utility of osteopontin determination in monitoring women with CA-125-negative disease is worthy of exploration. However, larger prospective trials will be needed to assess the ability of serum osteopontin to provide diagnostic and prognostic information or indications of treatment response.

